# Novel variant in *CADM3* causes Charcot–Marie–Tooth disease

**DOI:** 10.1093/braincomms/fcad227

**Published:** 2023-09-05

**Authors:** Abdoulaye Yalcouyé, Adriana P Rebelo, Lassana Cissé, Lynette Rives, Salia Bamba, Joy Cogan, Kevin Esoh, Salimata Diarra, Kimberly M Ezell, Abdoulaye Taméga, Cheick O Guinto, Maike F Dohrn, Rizwan Hamid, Kenneth H Fischbeck, Stephan Zuchner, Guida Landouré

**Affiliations:** Faculté de Médecine et d’Odontostomatologie, USTTB, Bamako, Mali; Division of Human Genetics, Department of Pathology, University of Cape Town, Cape Town, South Africa; Dr. John T. Macdonald Foundation Department of Human Genetics, John P. Hussman Institute for Human Genomics, University of Miami Miller School of Medicine, Miami, USA; Faculté de Médecine et d’Odontostomatologie, USTTB, Bamako, Mali; Department of Pediatrics, Vanderbilt University Medical Center, Nashville, USA; Faculté de Médecine et d’Odontostomatologie, USTTB, Bamako, Mali; Department of Pediatrics, Vanderbilt University Medical Center, Nashville, USA; Division of Human Genetics, Department of Pathology, University of Cape Town, Cape Town, South Africa; Faculté de Médecine et d’Odontostomatologie, USTTB, Bamako, Mali; Neurogenetics Branch, National Institutes of Neurological Disorders and Stroke, Bethesda, USA; Department of Pediatrics, Vanderbilt University Medical Center, Nashville, USA; Faculté de Médecine et d’Odontostomatologie, USTTB, Bamako, Mali; Faculté de Médecine et d’Odontostomatologie, USTTB, Bamako, Mali; Service de Neurologie, Centre Hospitalier Universitaire Point ‘G’, Bamako, Mali; Dr. John T. Macdonald Foundation Department of Human Genetics, John P. Hussman Institute for Human Genomics, University of Miami Miller School of Medicine, Miami, USA; Department of Neurology, Medical Faculty RWTH Aachen University, Aachen, Germany; Department of Pediatrics, Vanderbilt University Medical Center, Nashville, USA; Neurogenetics Branch, National Institutes of Neurological Disorders and Stroke, Bethesda, USA; Dr. John T. Macdonald Foundation Department of Human Genetics, John P. Hussman Institute for Human Genomics, University of Miami Miller School of Medicine, Miami, USA; Faculté de Médecine et d’Odontostomatologie, USTTB, Bamako, Mali; Neurogenetics Branch, National Institutes of Neurological Disorders and Stroke, Bethesda, USA; Service de Neurologie, Centre Hospitalier Universitaire Point ‘G’, Bamako, Mali

**Keywords:** CMT, next-generation sequencing, *CADM3*, novel variant, Mali

## Abstract

*CADM3* has been recently reported causing a rare axonal Charcot–Marie–Tooth disease in three independent Caucasian families carrying a recurrent change. We describe the first alternative causative mutation in *CADM3* in a family from black African and also observed *de novo* in a patient of Caucasian ancestry. The disease inheritance was consistent with autosomal dominant and sporadic patterns, respectively. Eight patients and their relatives were enroled from both families. The mean age at diagnosis was 33.9 years, and walking difficulty was commonly the first symptom. Neurological examination showed distal muscle weakness and atrophy, sensory loss and foot and hand deformities. A high clinical variability was noted, but as seen in *CADM3*-associated neuropathy, symptoms were more pronounced in the arms in some patients. Nerve conduction studies showed no response in most of the examined nerves, and an axonal type of neuropathy, where recorded. Whole exome sequencing revealed a novel missense variant (c.1102G>T; Gly368Cys) in *CADM3*, segregating with the disease. Functional analyses showed a significant decrease in CADM3-Gly368Cys protein levels in the membrane and major structural changes in its predicted secondary structure. Therefore, we extend the genotype spectrum of C*ADM3*, underlining the need for genetic studies in underrepresented populations like in Africa.

## Teaser

Yalcouyé *et al*. report that variants in *CADM3* gene cause an axonal-type neuropathy. This study provides further evidence of the implication of *CADM3* in the pathogenesis of Charcot–Marie–Tooth disease and raises the importance of including diverse and underrepresented populations like African in genetic studies.

## Graphical Abstract

**Graphical Abstract:**
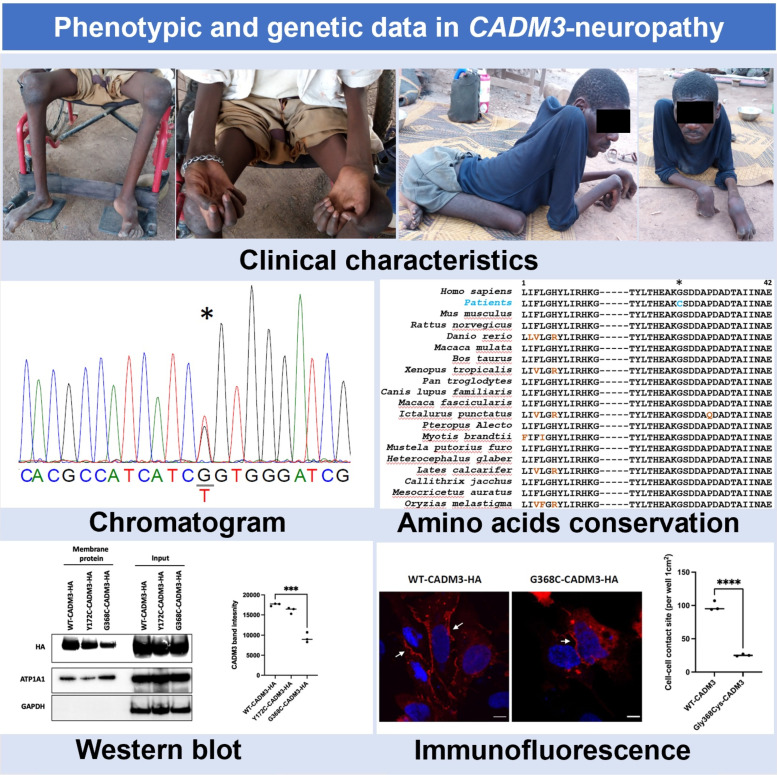

## Introduction

Charcot–Marie–Tooth disease (CMT) is the most common hereditary peripheral neuropathy with a high clinical and genetic heterogeneity. Characterized by progressive, distally ascending motor and sensory loss and feet deformities, its subtypes are broadly subclassified into demyelinating (CMT1 and CMT4) and axonal forms (CMT2).^1-3^ The genetic heterogeneity of axonal CMT makes the targeted gene testing strategy less efficient, especially in understudied populations such as African.^1^ Over the past few decades, the advent of next-generation sequencing has led to the identification of nearly 100 different genes.^4,5^ However, only 20–30% of CMT2 have been genetically diagnosed,^6^ and a few genetically confirmed CMT cases have been reported in the sub-Saharan African population, including Mali.^7-11^ More recently, whole exome sequencing has allowed the identification of a novel CMT gene, *CADM3* (OMIM: 609743).^12^ *CADM3* encodes a member of the CADM family of cell–cell adhesion molecules. The subcellular distribution showed that CADM3 is highly enriched in the cell membrane. CADM3 is expressed in axons, where it interacts primarily with *CADM4* (OMIM: 609744) and plays an important role in axon guidance, myelination and maintenance of the axonal architecture.^12^ One recurrent *CADM3* variant (Tyr172Cys) causing CMT2FF (OMIM: 619519) has been identified in three unrelated Caucasian families, with marked motor and especially upper limb involvement. In this study, we report a second pathogenic variant in the *CADM3* gene in two unrelated families: one of a black African ancestry from Mali and another a Caucasian American with a CMT2 phenotype.

## Material and methods

### Ethical compliance

The study was conducted in full accordance with the Declaration of Helsinki and was approved by the institutional ethics committee of the Faculty of Medicine and Dentistry of Bamako, Mali (N°2020/129/CE/FMOS/FAPH). Informed consent and assent for minor participants were obtained prior to their enrolment in this project, including the permission to publish photographs or videos.

### Clinical and neurophysiological assessment

All participants have undergone a careful clinical examination by a research team including a neurogeneticist and neurologists. Nerve conduction studies were performed in selected affected individuals.

### Genetic analyses

DNA was extracted from peripheral blood in all available family members using the QIAGEN Puregene Blood DNA kit C (Qiagen, Germantown, MD) following manufacturer’s instructions.

**Figure 1:**
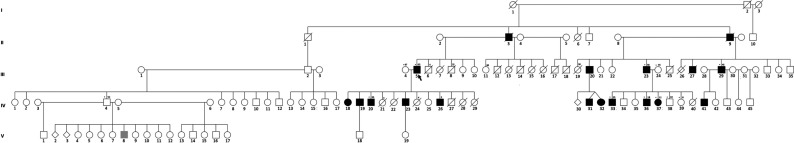
Pedigree of Family 1 showing a dominant inheritance pattern (arrow indicates the proband and asterisks the participants seen in clinic).

For whole exome sequencing, DNA samples of five individuals (III.4, III.5, III.29, IV.20 and IV.33) of Family 1 and one (III.2) of Family 2 were exome sequenced, and Fastq data were aligned to the human reference hg19 genome using a well-established bioinformatic algorithm and software (details are provided in Supplementary material). Putative variants were confirmed through Sanger sequencing, and segregation analysis was assessed in available individuals (Fig. 1A).

### Functional analyses

#### Protein modelling

Since there is no solved structure for CADM3 protein, the CADM3 isoform 1 (NM_021189.5) protein sequence (NP_067012.1) was retrieved from the NCBI GenePept database in FASTA format, and secondary structures were predicted for the wild-type (WT) as well as mutant sequences using the PSIPRED workbench (http://bioinf.cs.ucl.ac.uk/psipred). The 3D structures of CADM3 G368 WT and C368 mutant proteins were modeled on the Swiss Model server using the Alpha Fold-predicted CADM3 3D structure (https://alphafold.ebi.ac.uk/entry/Q8N126) as a template. The 3D structures were then refined using the Seok lab’s GalaxyRefine algorithm.^13^ The PyMol software was used for hydrogen bond analysis and structure visualization.^14^

#### Generation of constructs

Plasmid encoding the open reading frame of CADM3 (NM_021189.4) fused to an HA tag was obtained from GeneCopoeia. Site-directed mutagenesis was performed to generate plasmids with the novel mutation corresponding to Gly368Cys. The point mutation was introduced by polymerase chain reaction using the Q5 Site-Direct Mutagenesis kit (NEB) with the following primers: 5′-CGCCATCATCTGTGGGATCGTG-3′ and 5′-TGGTAGGTGCTGGAGGAG-3′.

#### Cell surface protein isolation and western blot

HEK293 cells were transfected with CADM3 plasmids, WT, Gly368Cys and Tyr172Cys using Lipofectamine 3000 (Thermo Fisher). The next day, cells were harvested for cell surface protein extraction using Pierce Cell Surface Protein Isolation Kit (Thermo Scientific) according to the manufacturer’s protocol. Plasma membrane proteins were biotinylated and extracted using the Pierce Cell Surface Biotinylation and Isolation Kit (Thermo Scientific) following the manufacturer’s protocol. Briefly, cells were incubated with the biotinylation reagent, Sulfo-NHS-SS-Bio, for 10 min. Cells were subsequently washed and lysed on ice for 30 min. Biotinylated proteins were captured with neutravidin resin for 30 min. Resin was washed, and proteins were eluted with dithiothreitol. Western blot was performed with the following antibodies: HA (3724, Cell Signaling), ATP1A1 (ab7671, Abcam) and GAPDH (sc-47724, Santa Cruz). Membranes were visualized using the FluorChem E FE0436 imaging system (ProteinSimple).

#### Immunofluorescence

We used human oesteosarcoma cell lines (U2OS cells) that were plated in an eight-well slide chamber (1 cm^2^ per well) and transfected with CADM3 plasmids. Cells were fixed with 4% paraformaldehyde for 15 min and permeabilized with cold methanol for 5 min. Cells were incubated with the monoclonal anti-HA primary antibody, Alexa Fluor 555 secondary antibody (Thermo Fisher) and 4′,6-diamidino-2-phenylindole (DAPI). Cells were imaged by confocal microscopy with a ×60 objective lens (Zeiss LSM710). Intercellular contact sites formed between two cells were counted using a tally counter for a total of 15 min for each well (three WT and three mutants) in a blinded experiment.

#### Statistical analysis

Student’s *t*-test was performed to determine the statistical significance of the mean difference of the quantitative data between two groups using Prism. A *P*-value less than 0.05 was considered statistically significant.

## Results

### Clinical phenotype

#### Family 1

**Figure 2:**
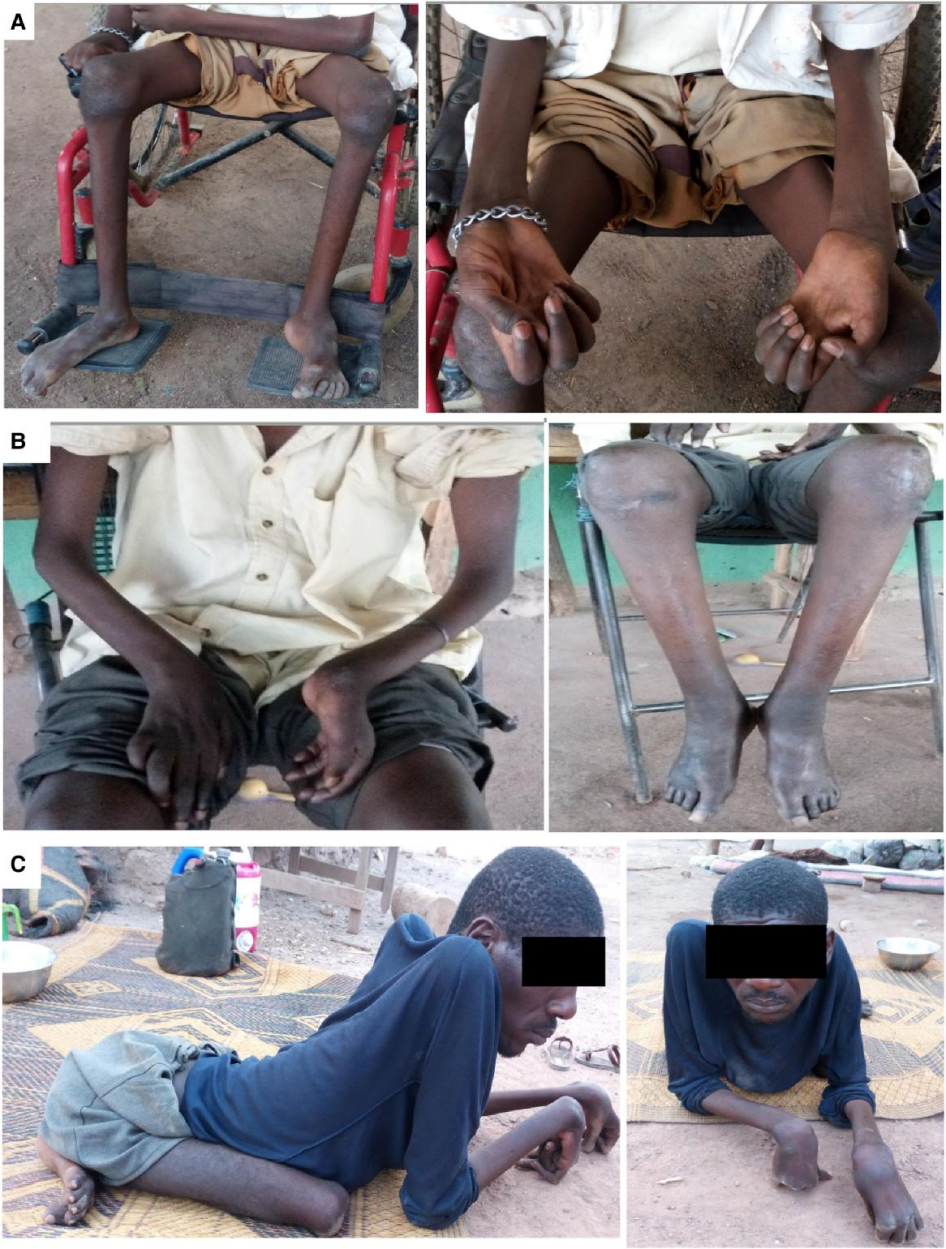
**Clinical findings.** (**A–C**) Images of the Patients IV.20, IV.36 and III.29 showing phenotypic variability between patients from different generations with foot and hand deformities and muscle atrophy and weakness.

Seven patients (six males and one female) and their relatives from a large family of Fulani ethnicity were enroled. The disease distribution was consistent with an autosomal dominant pattern of inheritance (Fig. 1). The mean age at diagnosis was 35.7 years, ranging from 12 to 65 years, and walking difficulty was the common starting symptom. The neurological examination showed distal muscle weakness and wasting and mild sensory impairment, steppage gait, decreased to absent tendon reflexes throughout, foot deformities (*pes cavus* and *planus*) and hammer toes in all affected individuals (Fig. 2A–C). An important clinical variability was noted within the family with some younger patients appearing to be more affected than the older ones (Fig. 2A and B). However, some patients had more pronounced upper limb involvement than lower limb symptoms (Fig. 2B and Video 1). One patient (IV.36) was wheelchair bound at age of 18 years, while his 50-year-old father (III.23) and the oldest enroled individual (III.4, 65 years) had mild symptoms. Moreover, besides having distal muscle weakness, one patient displayed a severe lower limb motor deficit likely due to the sequelae of poliomyelitis (Fig. 2C). In fact, parents reported that at age of 2 years, he presented an acute diarrhoeal syndrome with fever after which they noticed muscle weakness that worsened gradually. Nerve conduction studies were performed in five patients, and no response was recorded in most of the examined nerves, in severely affected individuals. However, where recorded, responses were consistent with an axonal type of neuropathy with reduced amplitude of the compound motor action potential and sensory nerve action potential while conduction velocities were in the normal range. All phenotypic and genotypic characteristics are summarized in Table 1.

**Table 1:** Phenotypic and genetic findings in patients with CMT2FF

						Clinical examination findings							Laboratory findings					
						Muscle weakness UL		Muscle weakness LL		Sensory loss	Tendon reflexes	Skeletal deformities	Nerve conduction studies					CADM3 variant
Patients	Age (years)	Sex	Age of onset (years)	CMTNSv2	First symptom	Proximal R/L	Distal R/L	Proximal R/L	Distal R/L				Median			Sural	Peroneal	c.1102G>T; Gly368Cys
													SNAP Amp	CMAP Amp	CV m/s	SNAP Amp	CMAP Amp	
F1/III.5	65	M	Fifth decade	6	Hand weakness	5/5	3/4	5/5	3+/4	Minimal	Decreased	*Pes cavus*, hammer toes	6	9.1	48	3	3.2	c.1102G>T; Gly368Cys
F1/III.23	50	M	Fourth decade	2	Muscle atrophy	5/5	5/5	5/5	5/5	Mild	Decreased	Absent	14	14.9	51	11	9	c.1102G>T; Gly368Cys
F1/III.29	45	M	First decade	16	Walking difficulty	5/5	0/0	5/5	0/0	Mild	Absent	*Pes planus*	5	NR	NR	NR	NR	c.1102G>T; Gly368Cys
F1/IV.20	35	M	First decade	22	Hand weakness	3/3	0/0	1/1	0/0	Mild	Absent	Pes cavus	NR	NR	NR	5	NR	c.1102G>T; Gly368Cys
F1/IV.33	25	M	First decade	10	Walking difficulty and hand weakness	5/5	3/3	5/5	3+/5	Minimal	Decreased	Absent	15	17	51	10	2.6	c.1102G>T; Gly368Cys
F1/IV.36	18	M	First decade	18	Walking difficulty	5/5	0/0	4/4	0/0	Mild	Absent	*Pes cavus*, hammer toes, claw hands	ND	ND	ND	ND	ND	c.1102G>T; Gly368Cys
F1/IV.37	12	F	Second decade	5	Walking difficulty	5/5	4−/4−	5/5	3+/3+	Mild	Absent	*Pes cavus*	ND	ND	ND	ND	ND	c.1102G>T; Gly368Cys
F2/III.2	9	M	First decade	13	Foot deformity	4−/4−	3/3	3/3	2/2	Absent	Absent	*Pes valgus*						c.1102G>T; Gly368Cys

Muscle weakness based on Medical Research Council Scale (0–5). Note: CMTNSv2 was estimated only based on clinical signs and symptoms and did not include electrophysiological items. Amp, amplitude; SNAP, sensory nerve action potential; CMAP, compound motor action potential; CMTNSv2, Charcot–Marie–Tooth Neuropathy Score version 2; F, family; UL, upper limb; LL, lower limb, R, right; L, left; CV, conduction velocity; NR, no response; ND, not done; m/s, metre per second.

#### Family 2

A 9-year-old male patient from an American Caucasian family and his unaffected parents were enroled. Patient was born at term following an unremarkable pregnancy and without perinatal complications. The disease distribution in the family was suggestive of a sporadic case (Supplementary Fig. 1). He had a normal early development until around 2 years of age. He was noted to have progressive valgus deformity of the feet initially improved with bracing but followed by a progressive decline in gait stability and strength at around 3 years of age accompanied by weight loss, a proximal muscle weakness and atrophy, without significant sensory or pain complaint. He had reduced reflexes in upper limbs but did not present clinically apparent sensory abnormalities. Nerve conduction studies showed a motor-predominant, primarily axonal neuropathy. Right sural nerve biopsy was unrevealing, and quadriceps muscle biopsy showed Type 2 fibre predominance and Type 2 atrophy.

### Genetic data

**Figure 3:**
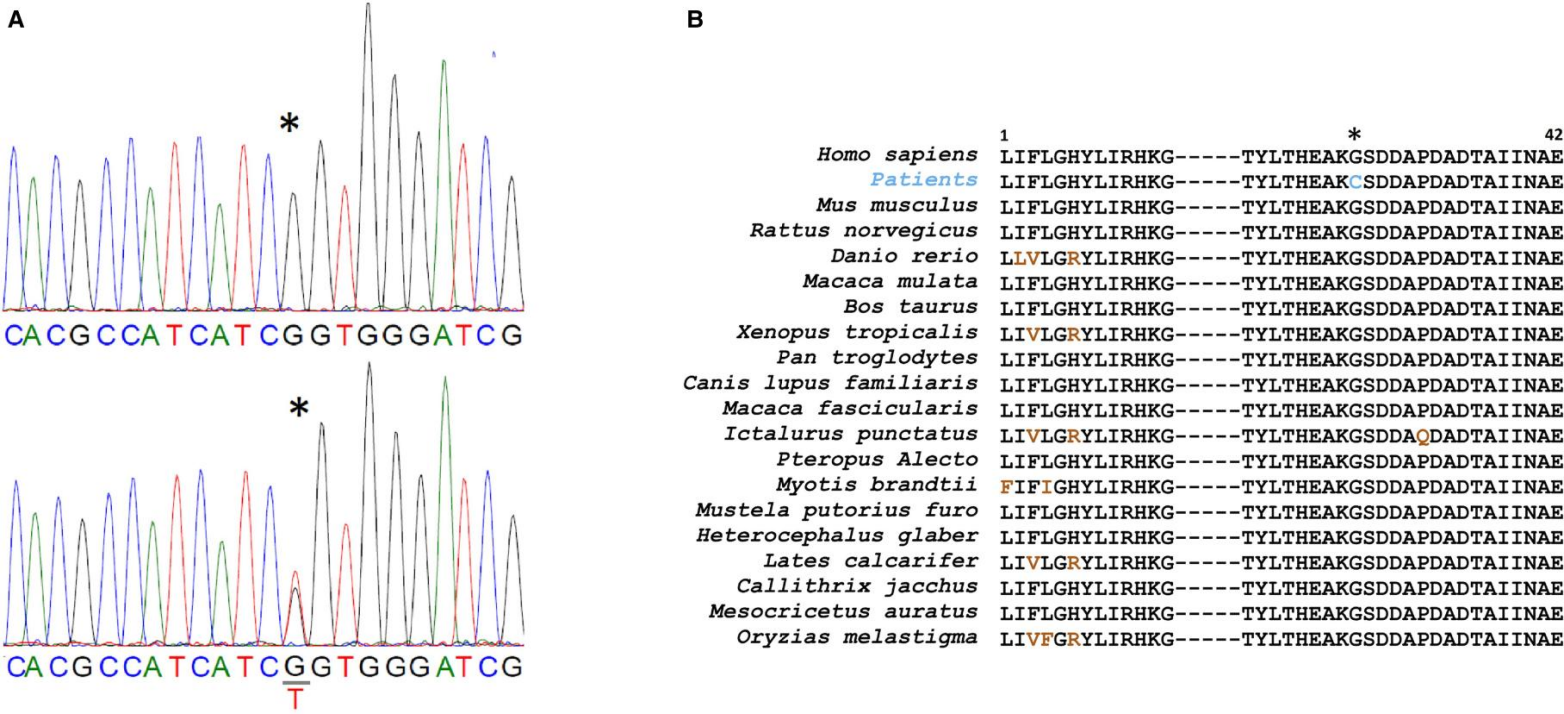
**Genetic data.** (A) Chromatogram showing the nucleotide G to T change (asterisk). (B) Amino acid conservation across a wide range of species.

Whole exome sequencing revealed a novel missense variant at position c.1102G>T, p.Gly368Cys, in the *CADM3* gene in both families. This variant was confirmed by Sanger sequencing in all 15 available members of the respective families (Fig. 3A) and was found to co-segregate with the disease. However, parents of the patient in the Family 2 did not have the variant, making it a *de novo* in that family. This variant is located in a highly conserved domain of the protein (Fig. 3B) with a phyloP100way score = 9.23 and is predicted to be deleterious using several *in silico* prediction tools including CADD score = 31 and SIFT = 0 (Supplementary Table 1). The variant is absent from gnomAD.

### Protein modelling

**Figure 4:**
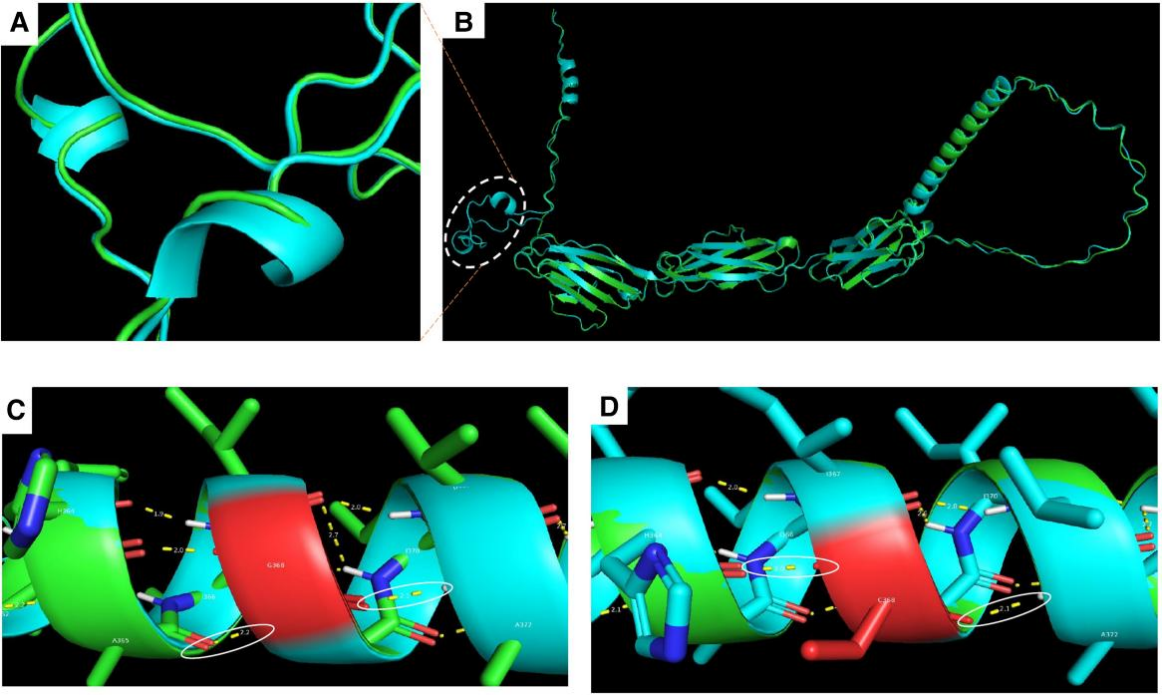
**Protein modelling.** (A, B) Superimposed 3D structures of CADM3 WT (green) and mutant (light blue) showing some regions with secondary structural changes (gain of helices in the mutant protein). (**C**, **D**) Hydrogen bond analysis of CADM3 WT and mutant protein structures. Hydrogen bonds involving the mutant site are indicated by the white circles. The protein cysteine residue has a free thiol group, which likely interferes with the insertion of the helix in the membrane. The WT G368 and mutant C368 formed two hydrogen bonds with histidine-364 and alanine-372 in a helical structure. The mutant C368 is a polar uncharged amino acid with a thiol (SH) side chain, which may affect the insertion of the helical structure into the membrane.

Several major structural changes were observed in the mutant. The helix structure at 6ASLL9 was completely changed to a beta strand, which was extended by two amino acids. In the predicted structures gained, there were two additional helices in the mutant structure that were not found in the WT structure (Fig. 4A and B). In addition, hydrogen bond analysis of the refined structures revealed that the WT Gly368 and mutant Cys368 formed two hydrogen bonds with histidine-364 and alanine-372 in a helical structure (Fig. 4C and D). However, this helical structure consists of residues forming a transmembrane helix (UniProt), meaning that it is supposed to be inserted in the membrane. Other minor changes were observed as depicted in the black boxes (Supplementary Fig. 2).

### Membrane isolation, western blot and immunofluorescence

**Figure 5:**
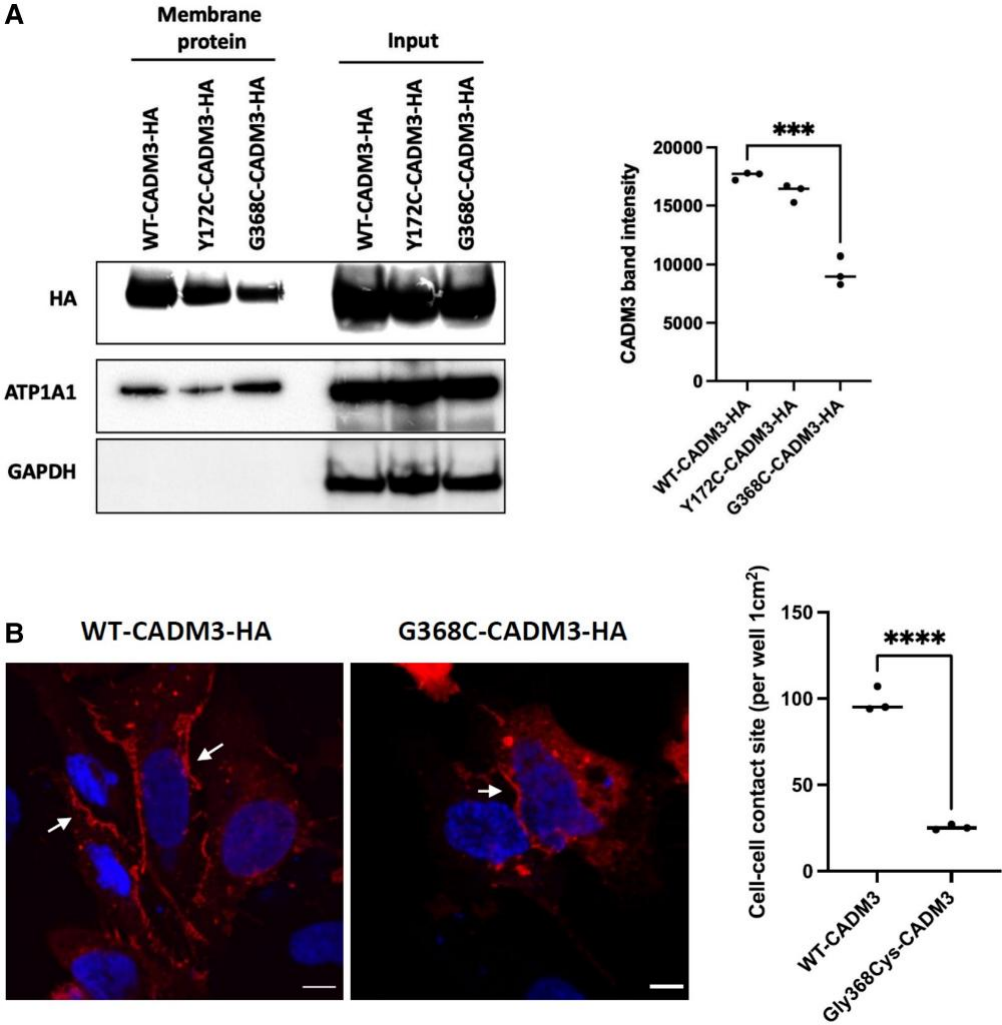
**Functional studies.** (A) Western blot analysis of membrane and cytoplasmic proteins extracted from HEK293-transfected cells showing that both WT and mutant proteins are predominantly enriched in the membrane compared to the cytoplasm, with a decrease level of the mutant. (**B**) Immunofluorescence of CADM3 (red) and 4′,6-diamidino-2-phenylindole (blue) in U2OS cells transfected with WT-CADM3-HA and G368C-CADM3-HA. White arrows indicate intercellular contact sites. Scale bars = 10 μm. Quantification of CADM3 staining cell–cell contact sites in WT and mutant (*P* < 0.001, two-tailed *t*-test, *n* = 3). The quantification of CADM3 staining at intercellular contact sites revealed an important decrease number of the mutant protein at contact sites compared to WT. This demonstrates that the mutant protein reaches the cell surface to a considerably lesser extent compared to the WT.

The newly identified CADM3 variant, Gly368Cys, is located in the protein transmembrane domain. Therefore, we decided to investigate whether the mutation affects membrane targeting. Cell surface proteins were isolated from HEK293 cells transfected with plasmids encoding the WT and mutants (Gly368Cys and Tyr172Cys). Western blot shows that both WT and mutant proteins are predominantly enriched in the cell surface (plasma membrane); however, reduced levels of membrane CADM3 were detected in the mutants compared to the WT (Fig. 5A; Supplementary Fig. 3A–C). We performed immunofluorescence to analyze protein co-localization and intercellular contact sites. Quantification of CADM3 staining at intercellular contact sites revealed a significantly decreased amount of the mutant protein at contact sites compared to WT (Fig. 5B). Our results demonstrate that the mutant protein reaches the cell surface to a considerably lesser extent compared to the WT. Interestingly, the variant Gly368Cys also creates a new cysteine, similar to the previously reported Tyr172Cys variant, which might interfere with the native disulphide bond important for the formation of the Ig-like loops.

## Discussion

CMT2 is a clinical and genetic umbrella of inherited peripheral neuropathies caused by primarily axonal dysfunction of the peripheral nerves. In contrast to CMT1, only one-third of CMT2 patients have a genetic diagnosis.^3,4^ *CADM3* is a recently identified CMT gene with the characteristic feature of upper limb involvement.^12^ The *CADM3* gene encodes a member of the CADM family of cell–cell adhesion molecules and plays an important role in axon guidance, myelination and maintenance of the axonal architecture.^12^ Pathological studies have shown that the removal of axonal Cadm3 promotes Schwann cell myelination in the *in vitro* dorsal root ganglion neuron/Schwann cell myelinating system in addition to its roles in axon.^15^ Furthermore, using an animal model, Rebelo *et al*.^12^ demonstrated that knockin mice carrying a heterozygous Tyr170Cys mutation in the *CADM3* gene (corresponding to the human Tyr172Cys mutation) showed a decreased expression of mutant Cadm3 in the sciatic nerve compared to controls and were consistent with abnormal axon–glia interactions. In this study, we investigated two families from different ethnic backgrounds, one of Fulani ethnicity from Mali and the other from American Caucasian descent with CMT2 phenotype, segregating the same pathogenic variant in *CADM3*. The clinical symptoms included distal muscle weakness and atrophy, foot and hand deformities, decreased or absent tendon reflexes and walking difficulties. Notably, a high phenotypic variability was seen within and between the families similar to the previous report.^12^ Some affected individuals showed more severe upper limb involvement when compared to the lower limbs. However, one patient in the Family 1 displayed a distinguishable phenotype with a pronounced segmental lower limb motor deficit, likely due to an overlap of CMT with poliomyelitis sequelae. In the first report of CMT2FF, an early onset of the disease was reported in three of the four patients with marked upper limb involvement. However, in one family, brisk reflexes suggested upper motor neuron involvement.^12^ In the families we report here, no symptoms of upper motoneuron involvement were noted, but the patient in Family 2 presented with foot deformities and a proximal weakness consistent with muscle involvement, pointing towards a clinical variability of CMT2FF. CADM3 is a cell adhesion molecule involved in maintaining the structure and function of cell junctions. Therefore, being membrane bound is an essential property of the protein. The variant described herein (Gly368Cys) is predicted to occur in a membrane-bound helix that is apparently crucial for the function of CADM3. The variant is a cysteine residue, and typically cysteine residues do not occur free in membrane proteins but rather form cysteine bridges.^16^ Therefore, the substitution to cysteine as a free residue might interfere with the function of CADM3 as a cell–cell adhesion molecule. More interestingly, western blot and immunofluorescence analyses showed that the mutant protein (Gly368Cys) reaches the cell surface to a considerably lesser extent compared to the WT protein. In addition, the variant Gly368Cys also creates a new cysteine, like the previously reported Tyr172C variant, which might interfere with the native disulphide bond important for the formation of the Ig-like loops.^12^

This is the second report of CADM3 neuropathy worldwide and the first that included a population of black ancestry. We present a comprehensive whole exome sequencing approach to describe the only second pathogenic variant in the *CADM3* gene causing CMT2. Our study provides further evidence of the implication of *CADM3* in the pathogenesis of CMT and raises the importance of collaborative studies including underrepresented populations like in Africa that can lead to the discovery of disease genes for more inclusivity in future therapeutic endeavour.

## Supplementary Material

fcad227_Supplementary_Data

## Data Availability

All data included in this study are available from the corresponding author upon reasonable request.
