# Supplementary material for: Novel variant in CADM3 causes Charcot–Marie–Tooth disease
Source: Brain Commun. 2023 Sep 5;5(5):fcad227. doi: 10.1093/braincomms/fcad227 (PMC10702457; doi:10.1093/braincomms/fcad227)

Supplementary materials

1- **Supplementary Materials and Methods**
2- **Supplementary tables**
3- **Supplementary figures**

Supplementary Materials and Methods

Whole exome sequencing and variants calling

For WES, DNA samples of five individuals III.4, III.5, III.29, IV.20, IV.33, were exome sequenced at Omega Bioservices (Norcross, GA, USA). The library preparation was performed with an Illumina Nextera Rapid Capture Exome Kit® (Illumina, San Diego, CA, USA) following the manufacturer's instructions. Fastq files generated from exome sequencing were aligned to the human reference hg19 genome using established bioinformatic algorithms and software (BWA-MEM, SAM tools) to generate SAM/BAM files. BAM files were processed to generate variant calling format (VCF) files in using GATK Tools. We then annotated the variants using Ensembl Variant Effect Predictor to include information regarding the gene, chromosomal coordinate(s), variants, type of mutation (frameshift, nonsense, nonsynonymous, splicing, and synonymous);16 prediction of the variant from multiple algorithms, allele frequencies in different databases including gnomAD, Exome Sequencing Project, dbSNP, 1000 Genomes, Complete Genomics, Exome Aggregation Consortium, and annotation of variants in clinical mutation database (ClinVar). Considering the variant annotated, the samples of the five individuals and pedigree/phenotype information corresponding, we used MendelScan v1.2.1 http://gmt.genome.wustl.edu/packages/mendelscan) to prioritize candidate variants based on the inheritance pattern, annotation, population frequency, and gene expression information. We finally considered variant based on the likelihood score ranging from 0 to 1 suggesting disease causing variant. Deleteriousness was verified with several in silico prediction tools including CADD, PolyPhen2, BayesDel addAF, DANN, DEOGEN2, EIGEN, FATHMM-MKL, LIST-S2, M-CAP, MutationAssessor, MutationTaster, PrimateAI, SIFT, SIFT4G, Provean, MutPred, LRT, MetaSVM, MetaRNN, REVEL

Supplementary table 1

Prediction tools	Variant
	c.1102G>T; Gly368Cys
CADD	31
PolyPhen2	Damaging
BayesDel addAF	Damaging
DANN	Damaging
DEOGEN2	Damaging
EIGEN	Pathogenic
FATHMM-MKL	Damaging
LIST-S2	Damaging
M-CAP	Damaging
MutationAssessor	Medium
MutationTaster	Disease causing
PrimateAI	Damaging
SIFT	Damaging
SIFT4G	Damaging
Provean	Damaging
MutPred	Damaging
LRT	Deleterious
MetaSVM	Damaging
MetaRNN	Damaging
REVEL	Pathogenic

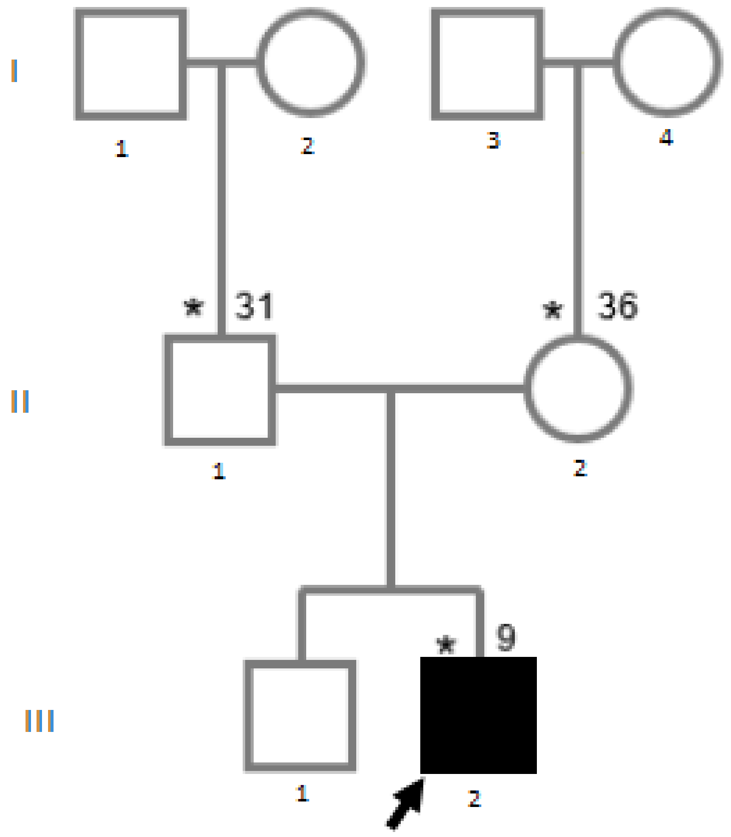

Supplementary figure 1: Pedigree of family 2. Showing a sporadic case, the black arrow indicates the proband, asterisks for those seen in clinics.

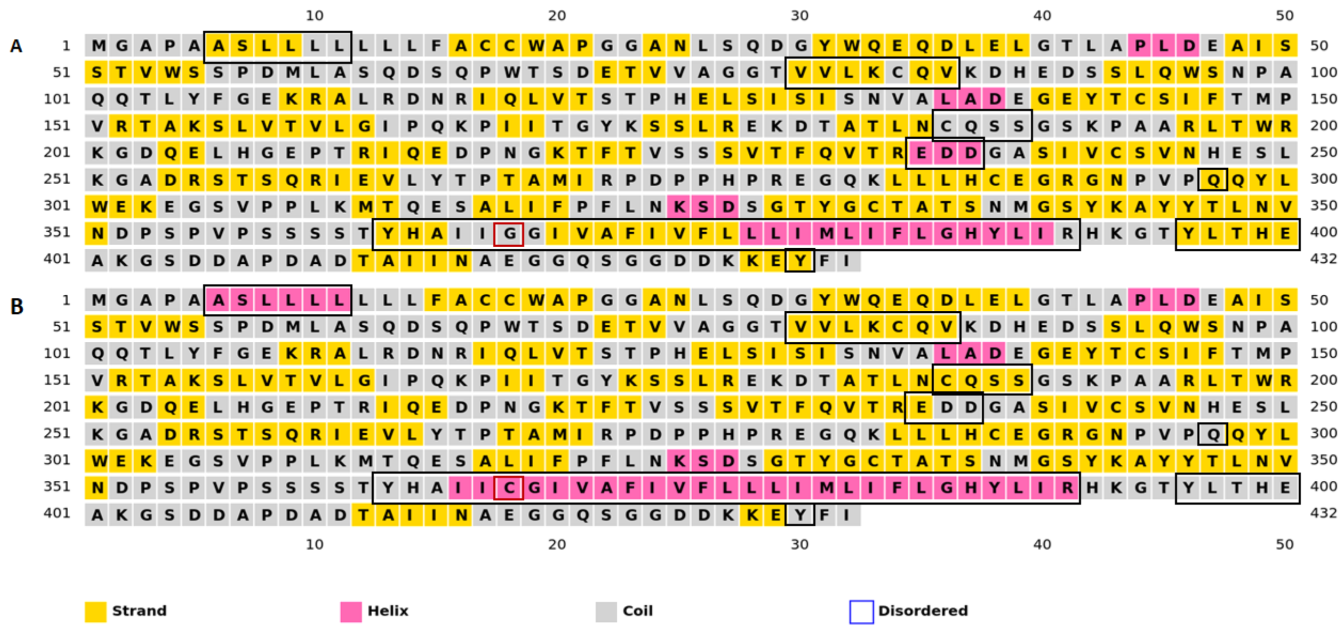

Supplementary figure 2: Secondary structure analysis. Wildtype A) and mutant B) CADM3 proteins. Red boxes show the site of the mutation, while black boxes show regions in which there are changes among the two sequences.

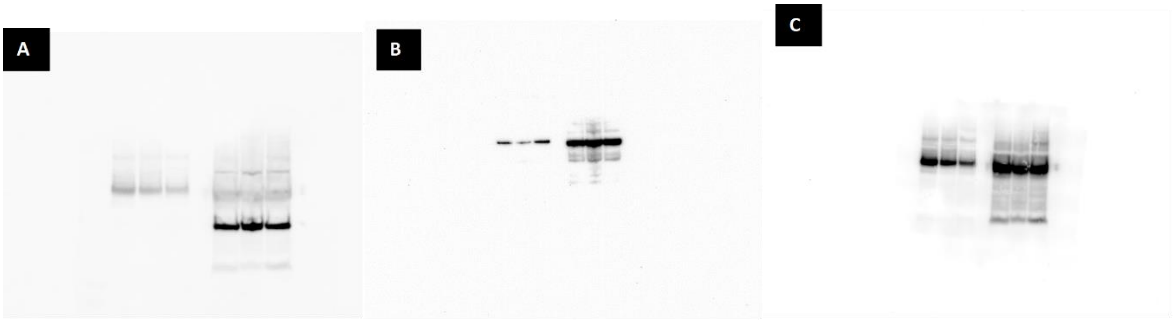

Supplementary figure 3: Uncropped images of the Western Blot images. A: GAPDH, B: ATP1A1_8bit and C: HA_8bit

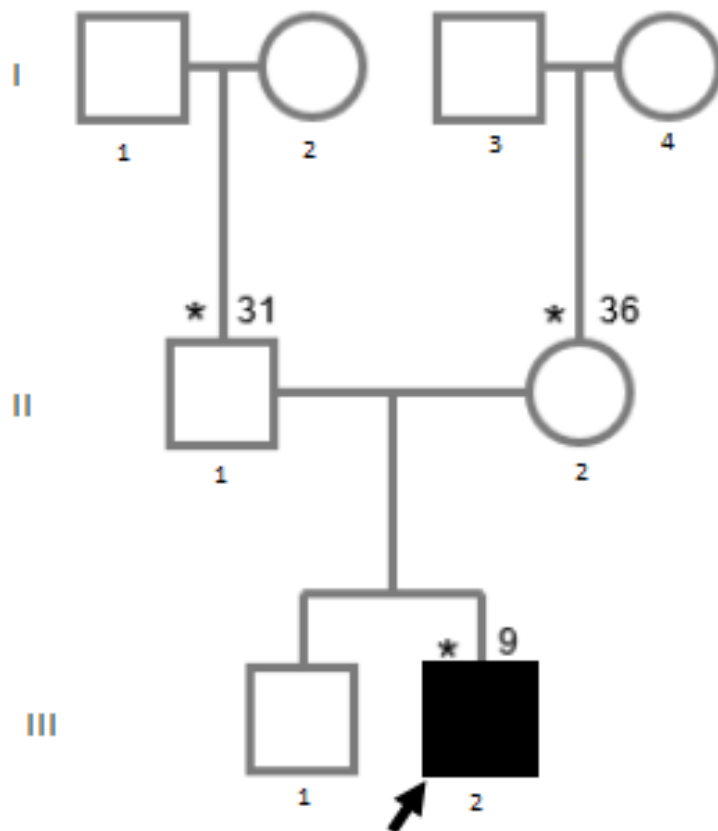

Supplement: fcad227_Supplementary_Data [file fcad227_Supplementary_Data.pdf]
